# Transcription factors regulating the fate and developmental potential of a multipotent progenitor in *Caenorhabditis elegans*

**DOI:** 10.1093/g3journal/jkac232

**Published:** 2022-09-05

**Authors:** Evan M Soukup, Jill C Bettinger, Laura D Mathies

**Affiliations:** Department of Pharmacology and Toxicology, Virginia Commonwealth University, Richmond, VA 23298-0613, USA; Department of Pharmacology and Toxicology, Virginia Commonwealth University, Richmond, VA 23298-0613, USA; Department of Pharmacology and Toxicology, Virginia Commonwealth University, Richmond, VA 23298-0613, USA

**Keywords:** SGP, transcription factor, multipotency, multipotent progenitor, RNAi, gonadogenesis, *Caenorhabditis elegans*

## Abstract

Multipotent stem and progenitor cells have the capacity to generate a limited array of related cell types. The *Caenorhabditis elegans* somatic gonadal precursors are multipotent progenitors that generate all 143 cells of the somatic gonad, including complex tissues and specialized signaling cells. To screen for candidate regulators of cell fate and multipotency, we identified transcription factor genes with higher expression in somatic gonadal precursors than in their differentiated sister, the head mesodermal cell. We used RNA interference or genetic mutants to reduce the function of 183 of these genes and examined the worms for defects in the somatic gonadal precursor cell fate or the ability to generate gonadal tissue types. We identify 8 genes that regulate somatic gonadal precursor fate, including the SWI/SNF chromatin remodeling complex gene *swsn-3* and the *Ci/GLI* homolog *tra-1*, which is the terminal regulator of sex determination*.* Four genes are necessary for somatic gonadal precursors to generate the correct number and type of descendant cells. We show that the *E2F* homolog, *efl-3*, regulates the cell fate decision between distal tip cells and the sheath/spermathecal precursor. We find that the FACT complex gene *hmg-4* is required for the generation of the correct number of somatic gonadal precursor descendants, and we define an earlier role for the *nhr-25* nuclear hormone receptor-encoding gene, in addition to its previously described role in regulating the asymmetric division of somatic gonadal precursors. Overall, our data show that genes regulating cell fate are largely different from genes regulating developmental potential, demonstrating that these processes are genetically separable.

## Introduction

As animals develop from a single-celled zygote, their cells transition through different states of developmental potential. Pluripotency is the capacity to generate all cell types of the mature organism. The expression of cocktails of transcription factors (TFs), called pluripotency factors, can induce differentiated cells to become pluripotent. The resulting induced pluripotent stem cells hold great therapeutic potential because they can be patient derived and have few ethical concerns (Deinsberger *et al.* 2020). Adult stem cells and lineage restricted progenitors are multipotent, meaning that they can generate a more limited array of cell types derived from a single lineage. Considerably less is known about the regulation of multipotency. One of the best-characterized multipotent stem cells is the hematopoietic stem cell (HSC), which gives rise to all blood cell types. Although differentiated blood cells have been converted into multipotent HSCs (Riddell *et al.* 2014), efforts to induce HSCs from unrelated cell types have been less successful (Esposito 2016). Genetic studies in model systems may reveal conserved strategies that give multipotent progenitors the capacity to produce diverse but restricted cell types.

The *Caenorhabditis elegans* somatic gonadal precursors (SGPs) are multipotent progenitors that generate all somatic cells of the gonad. In hermaphrodites, 2 SGPs produce 143 cells through 2 periods of cell division (Kimble and Hirsh 1979). During the first and second larval stages, each SGP generates 6 daughter cells—1 distal tip cell (DTC), 2 sheath/spermathecal (SS) precursors, 1 dorsal uterine (DU) precursor, 1 ventral uterine (VU) precursor, and 1 cell with the potential to become the anchor cell (AC). One of the bipotential AC/VU cells will differentiate as the AC, the other will become a VU precursor; this decision is determined by lateral signaling between the AC/VU cells (Kimble and Hirsh 1979; Greenwald *et al.* 1983; Seydoux and Greenwald 1989). The DTCs and AC differentiate into specialized signaling cells: the AC induces formation of the vulva (Kimble 1981) and DTCs signal to the underlying germ cells to promote their proliferation (Kimble and Hirsh 1979; Kimble and White 1981). The remaining progenitors divide again during the third and fourth larval stages to produce 140 cells. These cells form diverse tissues such as a contractile sheath that is important for ovulation, and the spermatheca and uterus, which are tubular epithelia that house sperm and developing embryos, respectively (reviewed in Hubbard and Greenstein 2000). Thus, the *C. elegans* SGPs produce diverse cell and tissue types through a completely defined cell lineage.

We have used the SGP/hmc cell fate decision as a paradigm for understanding how multipotency is regulated during development. The sisters of the SGPs are the left and right head mesodermal cells (hmcL and hmcR). These cells do not divide further; instead hmcR undergoes programmed cell death and hmcL terminally differentiates as the single head mesodermal cell (hmc), a cell of unknown function and neuron-like morphology (Sulston *et al.* 1983; Altun and Hall 2009). Therefore, the cell fate decision between SGPs and hmcs is one between multipotency and differentiation. We analyzed the transcriptomes of SGPs and hmcs and identified genes that are differentially expressed between these cell types (Mathies *et al.* 2019). Of particular interest to this study are genes with higher expression in SGPs than hmcs, referred to here as SGP-biased genes; among these were 184 genes encoding TFs. We expect that SGP-biased TFs will include regulators of SGP fate and multipotency.

We previously identified genes that are important for the SGP cell fate. *hnd-1* encodes a bHLH TF orthologous to *dHand* in vertebrates (Mathies *et al.* 2003), and 3 genes, *pbrm-1*, *swsn-1*, and *swsn-4*, encode subunits of a SWI/SNF chromatin remodeling complex (Large and Mathies 2014). *hnd-1* or SWI/SNF mutants have SGPs that express markers of the SGP and hmc cell fates, and they are frequently missing one or both gonadal arms in the adult (Mathies *et al.* 2003; Large and Mathies 2014). These mutant phenotypes suggest that SGPs are partially transformed into hmcs, and, as a result, they fail to produce mature gonadal cell types. Importantly, null mutations resulted in incompletely penetrant phenotypes, strongly arguing for redundancy in the regulation of the SGP/hmc cell fate decision.

To comprehensively search for regulators of SGP fate and multipotency, we examined the function of 183 SGP-biased TF genes using RNAi. Eight genes are important for normal expression of cell fate markers in SGPs, indicating that they play a role in the SGP/hmc cell fate decision. Notable for their strong phenotypes are the SWI/SNF complex gene *swsn-3* (Large and Mathies 2014) and the sex determination gene *tra-1* (Hodgkin 1987; Zarkower and Hodgkin 1993). Four genes are necessary for the production of the correct number and type of SGP descendants at the L3 larval stage; these genes are outstanding candidates for the regulators of multipotency in SGPs. We describe an earlier function for the nuclear hormone receptor (NHR) gene *nhr-25*, in addition to its role in regulating the asymmetric division of SGPs (Asahina *et al.* 2006), and we identify new roles for the FACT complex gene *hmg-4* and the E2F gene *efl-3* in somatic gonad development. Importantly, the genes regulating cell fate and multipotency are largely nonoverlapping, indicating that the genetic controls of cell fate and multipotency are distinct.

## Materials and methods

### Strains

Animals were grown at 20°C. All strains were derivatives of Bristol strain N2 (Sulston and Horvitz 1977). The following alleles and transgenes were used in this study and are described in *C. elegans II* (Hodgkin 1997) or cited references. *LGI: rdIs7* [*hnd-1::rde-1*] (Large and Mathies 2014) and *arTi361* [*gfp(flexon)::h2b*] (Shaffer and Greenwald 2022). *LGII: rdIs35* [*ehn-3::tdTomato*] (Mathies *et al.* 2019), *ccIs4444* [*arg-1::GFP*] (Kostas and Fire 2002), and *znfx-1(gg561)* (Wan *et al.* 2018). *LGIII*: *tra-1(e1099)* and *unc-119(ed3)* (Maduro and Pilgrim 1995). *LGIV*: *lin-22(ot287)* (Doitsidou and Hobert 2019). *LGV*: *rde-1(ne219)* (Tabara *et al.* 1999), *ceh-75(gk681)* (*C. elegans* Deletion Mutant Consortium 2012), *him-5(e1490)*, *qIs70* [*lag-2::YFP*] (Kidd *et al.* 2005), and *syIs51 [cdh-3::CFP]* (Inoue *et al.* 2002). *LGX*: *arTi237* [*ckb-3p::Cre*] (Shaffer and Greenwald 2022). Dominant GFP balancer for *LGI* and *LGIII*: *hT2[qIs48].*

### SGP-biased transcription factors

We queried the list of 2,749 SGP-biased genes (FDR ≤ 0.01, fold change ≥ 2) (Mathies *et al.* 2019) against the worm TF databases (Reece-Hoyes *et al.* 2005, 2011) and included any gene that was present in either database. In total, 184 of the SGP-biased genes were predicted to encode TFs (Supplementary File 1). Three of these genes, *lin-22*, *ceh-75*, and *znfx-1*, did not have available RNAi clones. We obtained loss-of-function alleles for each of these genes and crossed *rdIs35* [*ehn-3::tdTomato*] and *ccIs4444* [*arg-1::GFP*] into the mutant backgrounds. *znfx-1* is located on the same chromosome as *rdIs35* [*ehn-3::tdTomato*] and *ccIs4444* [*arg-1::GFP*]. We attempted to recombine the reporters with *znfx-1(gg561)* and were unsuccessful; therefore, this allele was only screened in our L4 screen. The only gene we were unable to screen was *nhr-129* because the single available RNAi clone did not grow and there were no loss-of-function alleles affecting only this gene. In total, we screened 183 of the 184 predicted SGP-biased TFs.

### Feeding RNAi

RNAi by feeding was performed essentially as described in Kamath *et al.* (2001). RNAi clones were obtained from a nearly complete TF RNAi library (MacNeil *et al.* 2015) or the commercially available RNAi libraries (Kamath *et al.* 2003; Rual *et al.* 2004). The clones were first streaked onto LB plates containing 50 µg/ml carbenicillin and 12.5 µg/ml tetracycline. Liquid LB cultures containing 50 µg/ml carbenicillin were inoculated from a single colony and grown for 16–18 h at 37°C. Nematode growth medium (NGM) plates containing 25 µg/ml carbenicillin and 1 mM IPTG were seeded with 150 µl of bacterial culture and incubated at 20°C for 2–3 days before worms were placed on the plates. Any RNAi clone that produced a phenotype in the primary screen was sequenced to ascertain that the clone was correct. Two clones were found to have different inserts than annotated and one was found to have mixed sequence indicative of either multiple plasmids or deletion within the insert; each of these clones was replaced with clones from other RNAi libraries, sequenced, and screened again.

### L1 screen

L4-staged worms were placed on the RNAi plates and allowed to develop for 36–48 h. The resulting adult worms were transferred to new RNAi plates and allowed to lay eggs for 1 h. Two collection windows were generated for each RNAi. The F1 progeny were screened approximately 24 h later, when they were mid-L1-staged larvae, using fluorescence and differential interference contrast (DIC) microscopy. Approximately 20 L1-staged worms were observed for the initial screen. The empty RNAi vector control was included with every batch. We recorded the number of cells in the gonad, expression of tdTomato in SGPs or anywhere outside of the gonad, and expression of GFP fluorescence in the gonad and the hmc. For GFP, we noted any expression that was brighter than that observed in the empty vector control. We also noted any cellular morphology changes. We performed a secondary screen for any genes having: (1) ≥ 25% of SGPs with GFP expression, (2) GFP expression in SGPs that was brighter than the control, or (3) any difference in the number of tdTomato-expressing cells in the gonad. We followed a similar protocol for the secondary screen, except we scored a minimum number of 40 L1-staged worms, and we noted 3 levels of GFP expression in the SGPs: dim, distinct, or bright.

### L4 screen

L4-staged worms were placed on the RNAi plates and allowed to develop for 36–48 h. The resulting adult worms were transferred to new RNAi plates and allowed to lay eggs for ∼24 h. The F1 progeny were screened as L4 larvae using a dissecting microscope. At least 50 worms were examined for gonadogenesis defects. We scored missing gonadal arms, disorganized gonads with a central patch of gonadal tissue (white patch, WP), and absent gonads (gonadless, Gon). Non-gonadal phenotypes were noted if observed. We performed a secondary screen using GS9401: *arTi361* [*gfp(flexon)::h2b*]; *arTi237* [*ckb-3p::Cre*] to mark all SGP descendants (Shaffer and Greenwald 2022). RNAi was performed as for the L4 screen, except the adult worms were allowed to lay eggs for 1 h and 2 collection windows were taken. The F1 progeny were screened approximately 36 h later, at or shortly after the L2/L3 molt, using fluorescence and DIC microscopy. Approximately 50 worms were scored for each RNAi knockdown and the number and organization of SGP descendants was recorded. We performed a follow-up screen on *efl-3* and *nhr-25* RNAi using markers for DTCs and the AC. For this purpose, we recombined *qIs70[lag-2::YFP]* and *syIs51[cdh-3::CFP]* to make RA344; this strain expresses YFP in DTCs and CFP in the AC. RNAi was performed as for GS9401, except the worms were allowed to develop for 48 h and were examined as early L4 larvae. To perform larval RNAi for *nhr-25*, we bleached RA344 worms and allowed the progeny to hatch in the absence of food. The resulting synchronized populations of L1 worms were plated on *nhr-25* or control RNAi bacteria and scored as early L4 larvae.

### Tissue-specific RNAi

For RNAi clones that produced an embryonic or larval lethal phenotype, we repeated the RNAi using a tissue-specific RNAi strain containing *rde-1(ne219)* and *rdIs7* [*hnd-1::rde-1*]. This strain rescues *rde-1* in mesodermal lineages including SGPs (Large and Mathies 2014). We crossed *rdIs35* [*ehn-3::tdTomato*] and *ccIs4444* [*arg-1::GFP*] into the tissue-specific RNAi background to create RA701. We crossed *arTi361* [*gfp(flexon)::h2b*] and *arTi237* [*ckb-3p::Cre*] into the tissue-specific RNAi background to create RA708. RNAi was performed as described above.

### *sex-1, tra-1*, and XO males

To examine XO males, we used a strain, RA481, that contains *rdIs35* [*ehn-3::tdTomato*] and *ccIs4444* [*arg-1::GFP*] in the *him-5(e1490)* background. The *him-5* mutation results in a higher rate of X chromosome nondisjunction and produces a higher incidence of males (Him) phenotype (Hodgkin *et al.* 1979). Males were distinguished from hermaphrodites by the size of the B cell nucleus. We crossed *rdIs35* [*ehn-3::tdTomato*] and *ccIs4444* [*arg-1::GFP*] into *tra-1(e1490)* and *sex-1(y263)* to create RA644 or RA705, respectively. *tra-1(e1490)* was balanced by the GFP-marked balancer *hT2[qIs48]*. *tra-1(e1490)* homozygotes were identified by the absence of GFP expression in the pharynx.

### Microscopy

Fluorescent reporters were visualized using an Axio Imager A1 microscope with a 63× Plan Apochromatic objective (Zeiss). Images were captured using an AxioCam MRm monochromatic camera with Zen software (Zeiss).

### Statistical analysis

Graphs were generated and statistical analysis was performed using Prism 9 version 9.3.1 (Graphpad). To analyze expression of the hmc marker in SGPs, we assigned a ranked numerical score for the level of expression (0 = none, 1 = “dim,” 2 = “distinct,” and 3 = “bright”) and compared each RNAi knockdown to the control using the Kruskal–Wallis test with Dunn’s post-hoc multiple comparisons test. The number of SGP descendants in each RNAi knockdown was compared to the control RNAi using 1-way ANOVA with Dunnett’s post-hoc multiple comparisons test.

## Results

We previously identified 184 predicted TF genes with higher expression in SGPs than hmcs (Mathies *et al.* 2019). Here, we used RNAi to examine the function of 183 of these genes to determine if they have roles in SGP fate or potential. For this screen, we have broadly defined SGP potential to include the production of the correct number and type of SGP descendants. We expect to identify genes that affect the SGP fate as well as genes that affect later cell fate decisions in the somatic gonadal lineage. We took a 2-pronged approach to screening. First, to identify genes that influence the SGP/hmc cell fate decision, L1 larvae were screened for differences in expression of SGP and hmc cell fate markers using fluorescence and DIC microscopy. Second, to identify genes that regulate SGP multipotency, L4 stage larvae were screened for gonadogenesis defects using a dissecting microscope. We reasoned that RNAi knockdown of genes required for multipotency would result in fewer or different gonadal cell types, which might lead to gross changes in gonadal morphology. For any genes that produced a lethal phenotype, the RNAi knockdown was repeated using an engineered tissue-specific RNAi strain that restricts RNAi to mesodermal lineages (Large and Mathies 2014). For genes without an RNAi clone, we examined available genetic mutants, where possible (see Materials and Methods).

### L1 RNAi screen identifies candidate SGP fate determinants

**Fig. 1.**
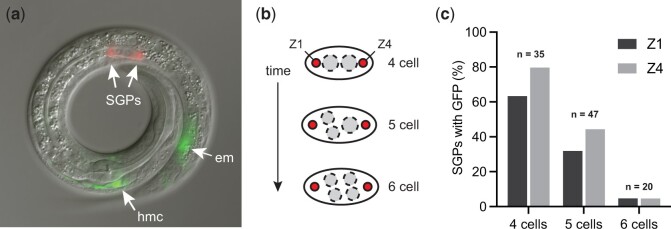
SGP and hmc markers. a) Expression of *arg-1::GFP* (green) and *ehn-3::tdTomato* (red) in an L1 animal. Fluorescent image overlay onto the corresponding DIC image. *ehn-3::tdTomato* is expressed exclusively in 2 somatic gonadal precursors (SGPs) within the gonad primordium; *arg-1::GFP* is expressed in the head mesodermal cell (hmc), an H-shaped cell near the pharynx in the head, and in enteric muscles (em) in the tail. b) Diagram of the gonad primordium over time. L1 worms hatch with a 4-celled gonad primordium containing 2 SGPs (Z1 and Z4) and 2 primordial germ cells (PGCs, dashed circles). The PGCs divide before either SGP divides, resulting in 4–6 cells in the gonad. c) Expression of *arg-1::GFP* in SGPs (Z1 and Z4) in wild-type worms with 4–6 cells in the gonad primordium.

We used a dual-color marker strategy to monitor the SGP and hmc cell fates (Fig. 1a). In wild-type worms at the L1 stage, *ehn-3::tdTomato* is expressed exclusively in the SGPs (Mathies *et al.* 2019) and *arg-1::GFP* is expressed in hmcs and enteric muscles in the tail (Kostas and Fire 2002; Zhao *et al.* 2007). The hmc has a distinctive H-shaped morphology and location (Altun and Hall 2009), making it easy to assess expression in hmcs. Since *arg-1::GFP* is expressed at low levels in SGPs (Large and Mathies 2014), we determined the best developmental window for scoring the SGP fate. We found that *arg-1::GFP* expression diminished over time such that worms with 6 cells in the gonad had minimal GFP expression in SGPs (Fig. 1, b and c).

We performed feeding RNAi on all of the SGP-biased TFs for which there were available RNAi clones (see Materials and methods). In order to minimize the background level of *arg-1::GFP* expression, only worms with 5 or 6 cells in the gonad were scored; most had 6 cells. First, we assessed expression of the SGP marker. Control animals almost always had 2 cells expressing the SGP marker in the gonad primordium (*n *=* *582/583 worms), as did most of the RNAi knockdowns. We never observed expression of the SGP marker in hmcs or anywhere outside of the gonad primordium. There were 12 genes for which RNAi knockdown resulted in occasional worms with fewer than 2 SGP marker-expressing cells in the gonad (Table 1). These are candidate SGP fate determinants.

**Table 1.** SGP-biased TFs with altered SGP marker expression.

	tdTomato expression in SGPs	
gene	No expression (%)	SGPs (*n*)
*nhr-92*	12	34
*tra-1*	6	34
*R05D3.3*	6	34
*F44E2.7*	6	34
*icd-2*	6	36
*moe-3*	5	44
*fkh-6*	3	30
*nhr-136*	3	34
*K11D12.12*	3	38
*ceh-63*	3	40
*zip-9*	2	42
*ztf-23*	2	42

Next, we examined expression of the hmc marker. We always observed expression in a cell with the correct location and morphology to be the hmc. Control RNAi worms also had dim hmc marker expression in 4.1% of Z1 cells and 5.7% of Z4 cells (*n *=* *583 worms). Many of the RNAi knockdowns resulted in a higher percentage of SGPs expressing the hmc marker. We chose 25% as a cutoff because it reflected 2 standard deviations from the mean for the control (Supplementary File 1). Using this criterion, we identified 19 genes that, when inactivated, resulted in a higher percentage of SGPs expressing the hmc marker or higher levels of hmc marker expression in at least 1 SGP (Table 2). These are candidate regulators of the SGP/hmc cell fate decision. In total, our primary screen identified 28 candidate genes.

**Table 2.** SGP-biased TFs with altered hmc marker expression.

	GFP expression in SGPs		
Gene	GFP in SGPs (%)	Brighter GFP (%)^a^	SGPs (*n*)
*tra-1*	79	41	34
*sex-1*	53	22	36
*ttx-3*	40	20	40
*nhr-120*	40	13	40
*ceh-40*	35	5	40
*T06G6.5*	30	0	40
*nhr-91*	29	0	38
*nfyb-1*	28	3	36
*blmp-1*	28	0	36
*mig-5*	25	11	36
*nhr-76*	25	0	40
*repo-1*	24	10	42
*swsn-3*	19	10	42
*moe-3*	18	7	44
*nhr-14*	18	8	38
*nhr-92*	21	6	34
*rnf-113*	12	5	42
*nhr-112*	5	3	38
*dhhc-4*	15	3	40

a GFP expression brighter than in the empty vector control.

**Fig. 2.**
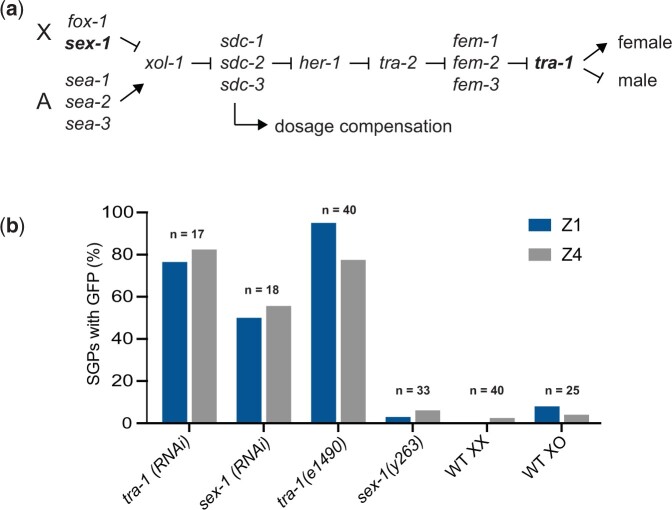
*tra-1* regulates the SGP/hmc cell fate decision. a) The *C. elegans* sex determination pathway. Arrows indicate positive regulatory relationships; bars indicate negative regulatory relationships. The ratio of the number of X chromosomes to autosomes (X: A) determines the activity of *xol-1* which is transmitted through a series of negative regulatory interactions to ultimately regulate the activity of *tra-1*. The *sdc* genes also regulate dosage compensation. *sex-1* and *tra-1* (bold) lie at opposite ends of the pathway. b) Expression of *arg-1::GFP* in SGPs of *tra-1* and *sex-1* RNAi or loss-of-function mutants (XX animals) compared to wild-type XX hermaphrodites and XO males.

Interestingly, 2 of our top candidate genes were members of the sex determination pathway. In *C. elegans*, sex is determined by the ratio of X chromosomes to autosomes, with XX individuals developing as hermaphrodites and XO individuals developing as males (Fig. 2a). *tra-1* is the terminal regulator of somatic sex determination; it is required for hermaphrodite development (Hodgkin 1987; Zarkower 2006). *sex-1* is one of several X chromosome signal elements required to promote hermaphrodite development (Carmi *et al.* 1998). Loss-of-function mutations in *tra-1* or *sex-1* result in XX animals developing as males. *sex-1* also controls dosage compensation; therefore the transformed XX animals frequently die because of inappropriate X chromosome gene regulation (Carmi *et al.* 1998; Gladden *et al.* 2007). We examined genetic mutants of *tra-1* and *sex-1* using the SGP and hmc markers and found that the strong loss-of-function mutation *tra-1(e1490)* caused significant hmc marker expression in SGPs, in agreement with our RNAi result, while the viable *sex-1(y263)* allele did not (Fig. 2b). We considered the possibility that males normally express *arg-1::GFP* in SGPs, and that *tra-1* mutant SGPs express the marker because they have a male fate. To test this idea, we examined *arg-1::GFP* expression in XO males and found that it is qualitatively similar to that of XX hermaphrodites (Fig. 2b), indicating that *tra-1* (and possibly *sex-1*) regulate the SGP/hmc cell fate decision.

**Fig. 3.**
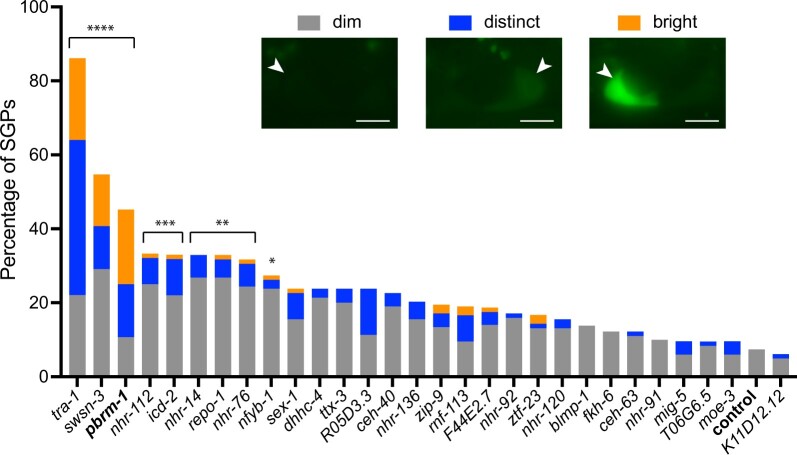
Genes affecting the expression of an hmc marker in SGPs. Expression of *arg-1::GFP* in SGPs. The gene being inactivated by RNAi is indicated on the X-axis; *pbrm-*1 and the empty vector control are in bold. The percentage of SGPs expressing dim (gray), distinct (blue), or bright (orange) GFP in SGPs is on the Y-axis. Statistical comparisons were made using the Kruskal–Wallis test with Dunn’s post-hoc tests to compare each RNAi to control. Statistical significance is indicated; *P ≤ 0.05; **P ≤ 0.01; ***P ≤ 0.001; ****P ≤ 0.0001. Insets show examples of dim, distinct, and bright expression. All images are 1.5 ms exposure with identical adjustment. Dim expression fades quickly and is difficult to capture. Scale bar is 5 µm.

We performed a secondary screen of all 28 candidate SGP fate regulators. The primary screen found differences in both the percentage of SGPs with hmc marker expression and the level of hmc marker expression in SGPs (Table 2). Therefore, in order to better classify our candidate SGP regulators, we recorded 3 levels of expression—dim (as seen in the control), distinct, and bright (Fig. 3). We included *pbrm-1*, a gene known to affect the SPG/hmc cell fate decision (Large and Mathies 2014), as a positive control. *pbrm-1* is not an SGP-biased TF because it is expressed in both SGPs and hmcs (Mathies *et al.* 2019). In control worms, we observed a small percentage of SGPs with dim hmc marker expression (7.4%, *n *=* *122 SGPs). By contrast, *pbrm-1(RNAi)* resulted in 45% of SGPs expressing the hmc marker and most had distinct or bright expression. RNAi of the candidate SGP fate regulators resulted in a wide distribution of hmc marker expression in SGPs (Fig. 3). Only 2 gene knockdowns, *tra-1* and *swsn-3*, had a higher percentage of SGPs with hmc marker expression than *pbrm-1*; both had distinct or bright expression. Interestingly, *swsn-3* encodes a subunit of SWI/SNF complexes that is predicted to be in a complex with PBRM-1. Of the remaining gene knockdowns, 6 had a statistically significant difference in expression of the hmc marker in SGPs, supporting the idea that the SGP/hmc cell fate decision is regulated by multiple TFs.

In contrast to the multiple TFs which when depleted increased hmc marker expression, we saw only minor effects on SGP marker expression. Control animals always had 2 SGPs with SGP marker expression, as did almost all of the RNAi knockdowns (Supplementary File 1). The exceptions were *mig-5*, *sex-1*, *nhr-136*, and *nhr-91*, which each had a single worm out of ∼40 with 1 or no cells in the gonad expressing the SGP marker; these worms had poor morphology by DIC making it difficult to assess the cause of the expression difference. In addition, we noted that *tra-1(RNAi)* resulted in worms with both SGPs positioned at the anterior end of the gonad; this phenotype is seen in *tra-1* loss-of-function mutants and results from the migration of Z4 toward the anterior of the gonad prior to its division (Mathies *et al.* 2004).

Taken together, our results indicate that several SGP-biased TFs are important for the SGP cell fate. These data further suggest that determination and/or maintenance of the multipotent SGP cell fate is driven by repression of the hmc terminal cell fate.

### L4 RNAi screen identifies candidate multipotency factors

We screened L4 staged worms using a dissecting microscope and looked for abnormalities in gonadal morphology, including missing gonadal arms, disorganized gonads, and absent gonads. Control RNAi worms always had a normal gonad (Table 3). Several RNAi knockdowns resulted in worms with a ventral “white patch” under the dissecting microscope. This phenotype is due to the presence of excess gonadal tissue near the vulva; it can result from a variety of somatic gonadal defects, including a failure to produce DTCs as is seen in Wnt pathway mutants (Siegfried and Kimble 2002; Siegfried *et al.* 2004). RNAi knockdown of *blmp-1*, *hmg-4*, and *repo-1* resulted in worms with missing gonadal arms. This phenotype can result from the absence of SGPs or the failure of SGPs to develop into gonadal arms (Mathies *et al.* 2003; Large and Mathies 2010, 2014). RNAi knockdown of *tra-1* resulted in a male-like gonad, as expected for *tra-1* loss of function (Hodgkin 1987). In total, our L4 screen identified 15 candidate SGP regulators (Table 3), 6 of which overlapped with our L1 screen.

**Table 3.** SGP-biased TFs with gonadal phenotypes.

gene	Gonadal phenotype	Other phenotypes^b^
Control	Wild type	None
*tra-1*	Male-like	Tra
*blmp-1*	Missing arm	Vab, Bmd
*cog-1*	White patch	Pvl
*fos-1*	White patch	
*efl-3*	White patch	Pvl
*egl-43*	White patch	delay, Pvl
*nhr-25*	White patch	delay, Let
*hmg-4*	Missing arm, white patch^a^	Emb
*swsn-3*	White patch^a^	Emb
*repo-1*	Missing arm, white patch^a^	Emb
*rnf-113*	White patch^a^	Emb
*sex-1*	White patch^a^	Dpy, Let, delay
*B0238.11*	White patch^a^	L1 arrest
*lpd-2*	White patch^a^	L3 arrest, Pvl
*C16A3.4*	White patch^a^	Emb, Let

a Tissue-specific RNAi was used to assess gonadal phenotype.

b Tra, transformed; Vab, variably abnormal; Bmd, body morphogenesis defect; Pvl, protruding vulva; delay, developmental delay; Let, larval lethal; Emb, embryonic lethal; Dpy, dumpy.

In order to more specifically identify genes that regulate the proliferation and developmental potential of SGPs, we performed a secondary screen using a GFP reporter that is expressed in all descendants of the SGPs (Shaffer and Greenwald 2022). We examined all genes that produced a phenotype in our L4 screen (Table 3), with the exception of *tra-1*, because the worms are transformed into males and the number of SGP descendants and organization of the gonad is different in males and hermaphrodites (Kimble and Hirsh 1979). We screened 5 additional genes, *nhr-112*, *icd-2*, *nhr*-*14*, *nhr-76*, and *nfyb-1*, that had significant expression of the hmc marker in SGPs at the L1 stage (Fig. 3) but appeared wild type in our L4 screen. We reasoned that these RNAi knockdowns might result in differences in the number or identity of SGP descendants that did not cause gross morphological defects at the L4 stage.

**Fig. 4.**
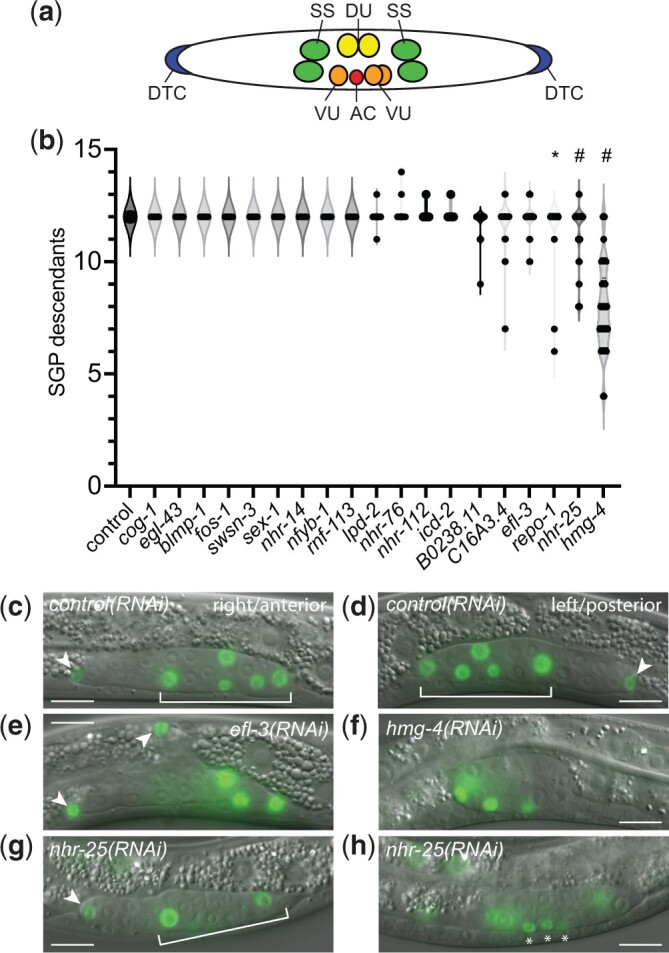
Genes affecting the number of SGP descendants at the L3 stage. a) Organization of the somatic gonad in early L3 larvae. Anterior is to the left; ventral is down. DTCs (blue) are located at the anterior and posterior poles of the gonad. Remaining somatic gonadal cells are centrally located with SS (green) precursors closest to DTCs, DU (yellow) and VU (orange) precursors more centrally located, and the AC located at the center of the gonad. b) Violin plot of the number of SGP descendants in early L3 larvae. The gene being inactivated by RNAi is indicated on the X-axis. Statistical comparisons were made using 1-way ANOVA with Dunnett’s post-hoc tests to compare each RNAi to the control. Statistical significance is indicated; *P ≤ 0.05; #*P* ≤ 0.0001. c–h) GFP fluorescence image overlay onto DIC images. GFP exposures are 200 ms. Scale bar is 10 µm. Arrowheads indicate DTCs; brackets indicate the somatic primordium (SPh) when present. (c and d) Empty vector RNAi control. The anterior arm (c) is on the right side of the worm; posterior arm (d) is on the left side of the worm. SPh is formed normally. e) *efl-3(RNAi)* results in extra DTCs that appear to develop in place of the dorsal SS cell. f) *hmg-4(RNAi)* results in fewer SGP descendants, no DTCs, and a round, underdeveloped gonad. Four SGP descendants are visible; 1 is out of the plane of focus. g and h) *nhr-25(RNAi)* results in worms with fewer central SGP descendants (g) and several small cells that are not organized into the SPh (asterisks).

We examined the worms early in the L3 larval stage, when the hermaphrodite gonad contains 12 somatic cells, and we counted the number of GFP-expressing cells in the gonad (Fig. 4, a and b and Supplementary File 1). Control worms always had 12 SGP descendants and the cells were correctly organized, with 2 DTCs at the poles of the gonad and the remaining 10 cells centrally localized in the SPh (Somatic Primordium of the hermaphrodite) (Fig. 4, b–d). Nine RNAi knockdowns also had 12 SGP descendants that formed an SPh, suggesting that, although these genes produced a phenotype at the L1 or L4 stage, they are unlikely to be important for SGP proliferation or multipotency.

RNAi targeting *hmg-4*, *nhr-25*, and *repo-1* caused a significant difference in the number of SGP descendants. The most extreme phenotype was seen in *hmg-4(RNAi)*, which had between 4 and 12 SGP descendants (8.0 ± 1.9, *n *=* *46). The SGP daughter cells were typically located at the periphery of the developing gonad (Fig. 4f), and DTCs were frequently absent. These observations are consistent with the ventral white patch phenotype that we observed at the L4 stage. *nhr-25(RNAi)* worms had between 8 and 13 descendants, and they displayed 2 predominant phenotypes. First, they had 2 DTCs with fewer central cells (Fig. 4g). Second, they had 1 or no DTCs, often accompanied by smaller cells that were not organized into the SPh (Fig. 4, g and h). These phenotypes are distinct from the previously described phenotype for *nhr-25(RNAi)* (Asahina *et al.* 2006; discussed later). *repo-1(RNAi)* had a low percentage of worms with 6 or 7 GFP-positive cells in the gonad (5.9%, *n *=* *51); these worms appeared to have descendants of only 1 SGP, judging by the positions and sizes of the daughter cells. We always observed 2 SGPs in *repo-1(RNAi)* L1s, suggesting that one of the SGPs failed to proliferate and produce the appropriate cell types. This phenotype is reminiscent of *hnd-1* or SWI/SNF mutants (Large and Mathies 2014). Finally, although it did not have a statistically significant difference in the number of SGP descendants, *efl-3(RNAi)* produced a highly penetrant and distinct phenotype. *efl-3(RNAi)* worms almost always had 3 or 4 DTCs in the gonad (89.1%, *n *=* *46); the extra DTCs were often seen in worms with 12 SGP descendants and appeared to result from a transformation of SS precursors into DTCs (Fig. 4e). Thus, our screen identified at least 4 genes that are important for generating the correct number and type of SGP descendants at the L3 stage, only one of which, *nhr-25*, had previously defined functions in the somatic gonad.

### *nhr-25* and *efl-3* are important for generating the correct number of DTCs

**Fig. 5.**
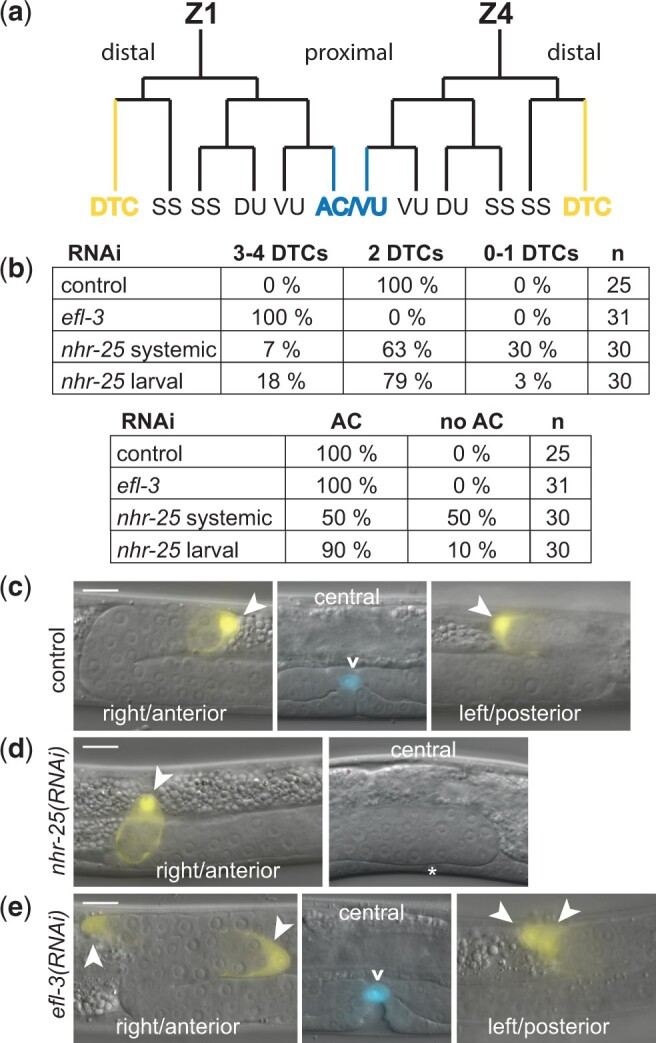
*nhr-25* and *efl-3* are required for the correct number of DTCs. a) SGP cell (Z1 and Z4) lineages. Each SGP produces 6 cells by the end of the L2 larval stage. The first division of Z1 and Z4 produces distal and proximal daughter cells with different fates: proximal daughters (Z1.p and Z4.a) make DU and VU precursors, an SS precursor (SS), and a cell that can become the AC (AC/VU, blue); distal daughters (Z1.a and Z4.p) make a DTC (yellow) and an SS cell. One of the AC/VU precursors becomes the AC; the cell that will adopt the AC fate is variable from animal to animal and determined by lateral signaling. b) Number of DTCs and ACs in control, *nhr-25*, and *efl-3* RNAi; *n* = number of worms. Systemic or larval RNAi is indicated for *nhr-25.* c–e) Expression of *lag-2::YFP* and *cdh-3::CFP* fluorescence. Fluorescent image overlay on DIC image. Left panel (right side, anterior); middle panel (central plane); right panel (left plane, posterior). YFP exposures are 200 ms; CFP exposures are 300 ms. Scale bar is 10 µm. Arrowheads indicate DTCs; carat indicates the AC. c) Control RNAi worms had DTCs at the leading end of the anterior and posterior gonadal arms and an AC above the developing vulva. d) *nhr-25(RNAi)* worms often had only 1 DTC at the leading end of a single gonadal arm (here, the anterior arm); they also frequently did not have an AC. Uninduced vulval precursor cell (asterisk). e) *efl-3(RNAi)* worms always had additional DTCs. Here, 4 DTCs sit at the ends of gonadal arms; 2 DTCs anterior and 2 DTCs posterior. Extra DTCs were present in worms with an AC.

The first division of the SGPs establishes the proximal distal axis of the gonad: central daughters adopt proximal fates and produce AC/VU precursors, while distal daughters adopt distal fates and produce DTCs (Fig. 5a). Wnt signaling and *nhr-25* act in opposition to determine proximal and distal fates in the gonad (Siegfried and Kimble 2002; Siegfried *et al.* 2004; Asahina *et al.* 2006), with the Wnt pathway promoting distal fates and *nhr-25* promoting proximal fates. Full proximal-to-distal cell fate transformation in *nhr-25(RNAi)* resulted in 4 DTCs and no AC (Asahina *et al.* 2006). In contrast to this published report, we did not observe extra DTCs in *nhr-25(RNAi)*; in fact, we often observed fewer than 2 DTCs. Interestingly, RNAi was performed differently in these experiments. The previous study used feeding RNAi starting with newly hatched L1 larvae (larval RNAi), whereas we used feeding RNAi beginning with the parental generation and continuing through the L3 stage (systemic RNAi). Systemic RNAi should reduce *nhr-25* mRNA at an earlier time in development, which may account for the earlier and distinct phenotype we observed.

To examine the phenotype of systemic *nhr-25(RNAi)* in more detail, we used a strain that contained markers for DTCs (*lag-2::YFP*) and the AC (*cdh-3::CFP*). We examined the worms as early L4 larvae, when both markers are expressed in the appropriate cell type. Control worms always had 2 DTCs and 1 AC (Fig. 5, b and c). By contrast, *nhr-25(RNAi)* worms had between 0 and 4 DTCs. The most common phenotype was a single gonad arm led by 1 DTC (Fig. 5d). Approximately half of the time, *nhr-25(RNAi)* worms had no AC as assessed by both marker expression and the absence of vulval induction. The absence of the AC was independent of the number of DTCs, suggesting that it was not simply the result of a proximal-to-distal cell fate transformation. These results are in contrast to *nhr-25(RNAi)* performed later in development, starting at the L1 stage, in which there were frequently worms with extra DTCs and rarely missing DTCs (Fig. 5b). We conclude that *nhr-25* is required for SGPs to produce the correct number and array of cell types, in addition to its previously defined role in specifying proximal gonadal cell fates.

To confirm that *efl-3(RNAi)* results in ectopic DTCs, we examined *efl-3(RNAi)* using the AC and DTC markers. We found that *efl-3(RNAi)* always resulted in extra DTCs and a single AC (Fig. 5b). Most of the worms had 4 DTCs and 1 AC (Fig. 5e), indicating that the extra DTCs did not result from proximal-to-distal cell fate transformation in the first SGP division. Instead, the extra DTCs appeared to result from the transformation of SS cells into DTCs, as evidenced by their location on the dorsal surface of the gonad (Fig. 4e), which is the normal location of SS precursors.

## Discussion

In this study, we conducted screens for regulators of cell fate and multipotency in a model multipotent lineage. We used RNAi to identify SGP-biased TFs that are important for the fate and/or developmental potential of SGPs. Seven genes were required for the expression of appropriate cell fate markers in SGPs but were dispensable for their subsequent development; these genes are likely to be involved in the determination or maintenance of the SGP fate. Three genes were required for the generation of the correct number and/or type of SGP descendants but did not have abnormal marker expression in SGPs; these genes are likely to include those that regulate the proliferation and/or developmental potential of SGPs, both of which are hallmarks of multipotency. Only a single gene affected both aspects of development, indicating that the regulation of cell fate and developmental potential are genetically separable.

### Redundancy in the regulation of the SGP/hmc cell fate decision

We previously identified HND-1 and components of the SWI/SNF chromatin remodeling complex as regulators of the SGP/hmc cell fate decision. Strong loss-of-function alleles of *hnd-1* or *pbrm-1*, a component of SWI/SNF complexes, displayed 2 incompletely penetrant phenotypes: (1) SGPs express markers for both the SGP and hmc cell fate, and (2) 1 or both of the gonadal arms fails to develop (Mathies *et al.* 2003; Large and Mathies 2014). We interpret these phenotypes as being the result of a partial cell fate transformation of SGPs into hmcs. Importantly, no single mutation, including strong loss of function and probable null alleles, is capable of fully transforming SGPs into hmcs, arguing for redundancy in the regulation of the SGP/hmc cell fate decision. Here we identified 8 additional genes for which RNAi knockdown resulted in expression of both SGP and hmc cell fate markers in SGPs.

Many of the genes we identified had only a modest effect on hmc marker expression, raising the possibility that SGP fate might be regulated by many genes, with each gene having a small effect. This is supported by our previous observations that null mutations in other SGP fate regulators cause incompletely penetrant phenotypes (Mathies *et al.* 2003; Large and Mathies 2014). However, there are important caveats to the interpretation of RNAi results. RNAi is known to produce incomplete gene inactivation and its efficacy varies from gene to gene and between tissue types. These limitations are reflected in genome-wide RNAi screens, which only detected ∼65% of genes with a previously reported phenotype (Kamath *et al.* 2003; Rual *et al.* 2004). Therefore, genes that had minor effects on marker expression by RNAi may produce a more severe and/or more penetrant phenotype using genetic mutants. In addition to the limitations of RNAi, our screen also would not be expected to identify genes with fully redundant functions. Indeed, we have previously described strong synergistic interactions between genes such as *hnd-1* and *ehn-3* (Mathies *et al.* 2003). Therefore, there are likely additional important regulators of the SGP/hmc fate decision that were not identified in this screen.

Only a single gene, *repo-1*, was required for normal expression of cell fate markers in SGPs and for the development of both gonadal arms. Therefore, *repo-1* falls into the same phenotypic class as *hnd-1* and SWI/SNF genes. *repo-1* encodes a homolog of SF3a66 which is a component of U2 snRNPs and is involved in pre-mRNA splicing (Takenaka *et al.* 2004). Very little is known about the function of *repo-1* in *C. elegans.* A semidominant allele of *repo-1* causes reversed polarity in the early embryo and loss-of-function alleles result in embryonic lethality without a polarity defect (Keikhaee *et al.* 2014). More recently, *repo-1* was found to be important for longevity and, in this context, it is thought to function as a splicing factor (Heintz *et al.* 2017). *repo-1* was included on the TF2.0 TF list because of its C_2_H_2_ zinc finger domain. C_2_H_2_ zinc fingers can bind RNA or DNA (Cassandri *et al.* 2017); therefore, it remains to be seen if REPO-1 acts as a TF or a splicing factor during SGP development.

Interestingly, most of the RNAi knockdowns that altered expression of cell fate markers in SGPs (Fig. 3) had little or no effect on the number of SGP descendants (Fig. 4). This observation strongly suggests that SGPs can recover from partial cell fate transformation to produce a normal somatic gonad. This is exemplified by *swsn-3(RNAi)*, which produced strong ectopic expression of the hmc marker in SGPs yet had no effect on the number or type of SGP descendants at the L3 stage. *swsn-3* encodes an accessory subunit of the SWI/SNF chromatin remodeling complex; therefore, it is predicted to function with *pbrm-1*, *swsn-1*, and *swsn-4* (Large and Mathies 2014). The biochemistry of the SWI/SNF complex has not been worked out in *C. elegans.* One possibility is that SWSN-3 is only present in SWI/SNF complexes in SGPs and not later during cell lineage progression. Alternatively, the effectiveness of RNAi may have been influenced by background differences between the strains used to assess these different phenotypes. The only existing *swsn-3* allele is viable and has no effect on gonadogenesis (Large and Mathies 2014), but because *swsn-3(RNAi)* causes embryonic lethality, it seems likely that this allele does not cause a strong loss of function. Clarification of the roles of *swsn-3* in somatic gonadogenesis will have to await the isolation of a strong *swsn-3* allele.

### *tra-1/GLI* is important for distinguishing SGPs from hmcs

A majority of *tra-1* mutant worms strongly expressed the hmc marker in SGPs, indicating that *tra-1* is required to suppress gene expression characteristic of a terminally differentiated cell in the multipotent SGPs. This expression difference cannot be explained by the sexual transformation of XX animals into males in *tra-1* mutants because wild-type (XO) males do not express *arg-1::GFP* in SGPs. We previously showed that *tra-1* regulates symmetry in the gonad primordium, independent of its role in sex determination (Mathies *et al.* 2004), and that this function is conserved in other nematode species (Kelleher *et al.* 2008). We have argued that these non-sex-specific functions of *tra-1* might represent the more ancestral function of *Ci*/*Gli* genes. First identified in *Drosophila*, Ci acts as the terminal TF in the Hedgehog (Hh) signal transduction pathway and is important for patterning in the embryo and imaginal discs (reviewed in Huangfu and Anderson 2006). Three vertebrate *Gli* genes have overlapping functions downstream of Sonic hedgehog (Shh) signaling and are important for central nervous system patterning and lung development (reviewed in Matise and Joyner 1999). In this light, the involvement of *tra-1* in the SGP/hmc cell fate decision might represent another example of *tra-1*’s more ancestral functions.

Our identification of *sex-1*, an upstream regulator of both dosage compensation and sex determination, hints at the possibility that *tra-1*’s function in the SGP/hmc cell fate decision might be regulated by the sex determination pathway. A viable *sex-1* allele did not replicate our RNAi result. However, this allele, *sex-1(y263)*, is a splice acceptor mutation that affects sex determination and dosage compensation; it is not a null allele (Gladden *et al.* 2007). Thus, it is possible that RNAi produces a stronger phenotype than the hypomorphic allele, and that this reveals a role for *sex-1* in the SGP/hmc fate decision. Alternatively, the *sex-1(RNAi)* phenotype may result from off-target effects of RNAi. *sex-1* encodes one of 284 NHRs in *C. elegans* (Antebi 2006); therefore, it is possibile that the *sex-1* RNAi phenotype is due to inactivation of another NHR. Our data clearly implicate *tra-1* in the regulation of the SGP fate decision. It will be interesting to see if *tra-1* is regulated in this context by the sex determination pathway.

### HMG-4 and the maintenance of cell fate

*hmg-4* encodes a subunit of the *C. elegans* FACT (facilitates chromatin transcription) complex. FACT is composed of 2 subunits, SSRP1 (structure-specific recognition protein 1) and SPT16 (Suppressor of Ty 16), and was identified for its role in promoting transcript elongation through nucleosomes (Orphanides *et al.* 1998, 1999). *Caenorhabditis elegans* FACT is required for normal cell cycle timing during embryogenesis (Suggs *et al.* 2018) and acts as a barrier to cellular reprogramming in adult tissues (Kolundzic *et al.* 2018). We found that *hmg-4(RNAi)* resulted in a reduced number of SGP descendants at the L3 stage and abnormal gonadal morphology at the L4 stage. Based on the absence of gonadal arm elongation, we infer that at least 1 cell type, the DTC, was absent in *hmg-4(RNAi)* worms. Therefore, *hmg-4* function is necessary for SGPs to generate the correct number and type of descendants, suggesting a role in SGP proliferation and multipotency. FACT functions as a barrier to reprogramming, in part, by maintaining cell fate. One possibility is that FACT maintains the SGP cell fate and, in its absence, the cells fail to execute their developmental program. FACT is also required for transcript elongation and results in longer cell cycle times. Alternatively, *hmg-4* might be required for proper execution of the cell division cycle during SGP development resulting in fewer SGP daughter cells.

### EFL-3 and cell fate

*efl-3* is one of 3 E2F-encoding genes in the *C. elegans* genome. It is most similar to the mammalian genes E2F7 and E2F8, and was identified for its ability to inhibit cell death in ventral cord neurons by promoting expression of the pro-apoptotic gene *egl-1* (Winn *et al.* 2011). It is also expressed in seam cell lineages and is required for development of the correct number of seam cells in the adult (Katsanos *et al.* 2021). We show here that loss of *efl-3* function in the somatic gonad results in additional DTCs. The supernumerary DTCs appeared to develop at the expense of the dorsal SS precursor, based on their position within the developing gonad and on the absence of dorsal SS cells in gonads with extra DTCs. The dorsal SS precursor is the sister of the DTC; therefore, a simple explanation is that *efl-3* is required for specification of the SS cell fate and/or inhibition of the DTC fate. E2F proteins are important regulators of the cell cycle, acting with DP to promote cell proliferation (Lam and La Thangue 1994). EFL-3 and its homologs, E2F7 and E2F8, lack important transactivation and interaction domains typically found in more canonical E2F proteins (Lammens *et al.* 2009). However, there is evidence to suggest that atypical E2F proteins act in cell cycle regulation. For example, human E2F7 promotes proliferation and inhibits differentiation of acute myeloid leukemia cells (Salvatori *et al.* 2012) and E2F8 overexpression promotes proliferation and tumorigenicity in breast cancer cell lines (Salvatori *et al.* 2012). SS cells continue to divide and generate multiple cell types, whereas DTCs are terminally differentiated. Therefore, another possibility is that EFL-3 is acting to promote proliferation in SS cells and that, in its absence, the cells differentiate into DTCs.

### *nhr-25* is a pleiotropic regulator of somatic gonad development

The first division of the SGPs is asymmetric and is governed by a Wnt/β-catenin signaling pathway that promotes distal fates. Mutations in Wnt pathway mutants result in all SGP daughter cells adopting a proximal fate; no DTCs are produced and the gonad does not elongate (Siegfried and Kimble 2002; Siegfried *et al.* 2004). *nhr-25* opposes Wnt signaling such that *nhr-25* loss of function results in extra DTCs at the expense of proximal cells (Asahina 2006). We found that *nhr-25* RNAi, when applied at an earlier stage in development, resulted in fewer DTCs and a loss of the AC. The simplest explanation for these different observations is that *nhr-25* acts early in SGPs and then again in opposition to Wnt signaling to determine proximal and distal fates. *nhr-25* acts in the epidermis to promote cell differentiation by repressing the expression of factors that promote a stem cell fate (Katsanos and Barkoulas 2022). It is hard to reconcile the *nhr-25* phenotype in the somatic gonad with a role in promoting differentiation. Instead, our results suggest that *nhr-25* promotes multipotency in SGPs: *nhr-25* loss of function does not affect the SGP cell fate but does affect the number and type of cells produced by SGPs. It will be interesting to compare the function of *nhr-25* in multipotent SGPs with that in multipotent epidermal stem cells.

## Supplementary Material

jkac232_Supplementary_Data

## Data Availability

Strains are available upon request. Supplementary File 1 contains all primary data from the RNAi screens. Supplemental material is available at G3 online.
